# Reply to “Matters arising: cost-effectiveness of first-line immunotherapy combinations with or without chemotherapy for advanced non-small cell lung cancer: a modelling approach”

**DOI:** 10.1186/s12885-024-12288-5

**Published:** 2024-07-22

**Authors:** Wen Hui, Zhixiang Gao, Min Zhu, Huazhang Wu, Yuanyi Cai

**Affiliations:** 1 Department of Science and Techonology, West China Hospital of Sichuan University, Chengdu, China; 2 Department of Pharmacy, Affiliated Central Hospital of Shenyang Medical College, Shenyang, China; 3 Department of Health Service Management, School of Health Management, China Medical University, Shenyang, China; 4 School of Medical Humanities Sciences, China Medical University, Shenyang, China

**Keywords:** Proportional hazard, Accelerated failure time model, Health utility

## Abstract

In this article, we read with great attention the correspondence by Bullement et al., regarding our published study on cost-effectiveness of first-line immunotherapy combinations with or without chemotherapy for advanced non-small cell lung cancer. We referred to a few the most important comments from Bullement et al. in our opinion, including proportional hazard (PH) assumption, accelerated failure time (AFT) model, and health utility, and made some explanations.

We read with great attention the correspondence by Bullement et al., regarding our published study on cost-effectiveness of first-line immunotherapy combinations with or without chemotherapy for advanced non-small cell lung cancer. We thank them for their interest in our work and for sharing the concerns about the methodology and interpretation of our findings. It is our honor to have this opportunity to learn from the world-leading experts in the subject area. We would like to make some explanations for a few the most important comments from Bullement et al. in our opinion, including proportional hazard (PH) assumption, accelerated failure time (AFT) model, and health utility.

First, testing for proportional hazard (PH) is an important step in the survival analysis. For example, using log-cumulative hazard plot mentioned in the Matters Arising, and it was also recommended in the National Institute for Health and Clinical Excellence (NICE) Decision Support Unit (DSU) technical support document 14 [1]. In our article, the model was based on a published network meta-analysis by Liu *et al* (2021) [2], which provided a constant for hazard ratio(HR) value. Usually, in each clinical trial, the HR is provided by authors, even if “crossing curve” occurs in the trial, like MYSTIC trial (NCT02453282) [3], which represents “point estimator (under a significance level)” or “average level”. Liu’ s work gave us a synthetized result. However, the issue of non-PH was not considered in their work. As the first phase of study, Liu’ s result gave us a reference, and we obtained an “overview” for incremental cost-effectiveness ratio (ICER) in our article. It appeared the limitation listed in the comments by Bullement et al. There is no doubt that constructing the “h(t) and HR(t)” for immunotherapies, using the method like fractional polynomial (FP) model by Jansen (2011) [4], Wiksten et al (2020) is more precise [5]. Through “HR(t)”, each ICER calculated in each time point will be “meaningful”. As the second phase of study, this work was already completed in our new research program in October 2023.

Second, when selecting the distributions to fit the reconstructed data, we have also considered the characteristics of distributions such as PH model or an accelerated failure time (AFT) model. Log-logistic distribution was one of nine basic distributions provided by R language after inputting “library(survHE)”.Because it was the AFT model, HR and Eq. 5 were not used in this phase, only the S(t) function was considered. The S(t) function of Log-logistic model was given in the framework of R programming language, which was used to fit the reconstructed data and to construct the partitioned survival model in the “heemod” package. The specific explanation was as follows:

If “help(Llogis)” was input, the following cumulative distribution function could be found (The specific description could be found in the part of “Note” of this help documentation.).

$$S\left(t\right)=\frac{1}{[1+{\left(\frac{t}{scale}\right)}^{shape}]}$$

In addition, the authors also highlighted the source of health utility and its value. The guidelines from the International Society for Pharmacoeconomics and Outcomes Research (ISPOR) and experts have pointed out the utility values can be synthesized to provide a pooled estimate, if they are sufficiently homogenous, and a strong justification should be provided for pooling utility values [6, 7]. However, considering the substantial heterogeneity of the pooling utility values in the previous meta-analysis, we used the health utility value sourced from an original survey. In our study, a Chinese utility value of 0.321 in the progressed disease health state sourced from an international research by Nafees *et al*. [2017] [8]. Prior economic evaluations from Chinese perspective also cited this value [9–11]. This value was lower than that mentioned by Bullement et al. This is because Nafees et al. uses time trade off (TTO) interviews with unaffected people to elicit public perceptions of living with progressive NSCLC. TTO can exaggerate the utility impact of progression compared to values derived directly from patients with the condition. There is no doubt that more suitable health utility value leads to more precise outcome, and we will seriously consider this in the future study.

The authors’ comments and recommendations related to the research methodology are valuable. The research was prepared from the year of 2021. All the explanations in this reply were just the description of the thought at that time point, and the limitations existed from the present point of view. The expert review of Bullement et al. makes a better conclusion, and we will improve upon this work in our next research.

## Data Availability

No datasets were generated or analysed during the current study.

## Abbreviations

PH: Proportional hazard
NICE: National Institute for Health and Clinical Excellence
DSU: Decision Support Unit
HR: Hazard ratio
ICER: Incremental cost-effectiveness ratio
FP: Fractional polynomial
AFT: Accelerated failure time
ISPOR: International Society for Pharmacoeconomics and Outcomes Research
TTO: Time trade off
